# A Comprehensive Technical Review of the Friction Stir Welding of Metal-to-Polymer Hybrid Structures

**DOI:** 10.3390/polym15010220

**Published:** 2022-12-31

**Authors:** Ali A. Barakat, Basil M. Darras, Mohammad A. Nazzal, Aser Alaa Ahmed

**Affiliations:** Mechanical Engineering Department, American University of Sharjah, Sharjah P.O. Box 26666, United Arab Emirates

**Keywords:** friction stir welding, hybrid structure, metal-to-polymer joining, process parameters, mechanical performance, microstructural characteristics

## Abstract

Metal–polymer hybrid structures are becoming desirable due to their wide range of applications in the automotive, aerospace, biomedical and construction industries. Properties such as a light weight, high specific strength, and design flexibility along with the low manufacturing costs of metal–polymer hybrid structures make them widely attractive in several applications. One of the main challenges that hinders the widespread utilization of metal–polymer hybrid structures is the challenging dissimilar joining of metals to polymers. Friction stir welding (FSW) shows a promising potential in overcoming most of the issues and limitations faced in the conventional joining methods of such structures. Several works in the literature have explored the FSW of different metal-to-polymer combinations. In some of the works, the joints are examined based on processing parameter optimization, microstructural characteristics, and mechanical performances. It is, therefore, important to summarize the findings of these works as a means of providing a reference to researchers to facilitate further research on the utilization of FSW in joining metals to polymers. Thus, this work aims to present a comprehensive technical review on the FSW technique for joining metals to polymers by reviewing the reported literature findings on the impact of materials, tools, process parameters, and defects on the strength and microstructure of the produced joints. In addition, this work reviews and presents the latest practices aiming to enhance the metal–polymer joint quality that have been reported in the literature.

## 1. Introduction

The demand on hybrid metal–plastic joints is on the rise due to their ability to create lightweight yet strong structures, making them attractive for the automobile, aerospace, electronics, and biomedical industries. For instance, the shift towards environmentally friendly lightweight materials in the automotive industry has led to an increase in the adoption rate of plastics [1]. Plastics consist of large molecules called polymers. Polymers have a high specific strength, low thermal expansion, and excellent fatigue and fracture strength. Moreover, polymers are light and easily formable materials. These properties make polymers an attractive material for the automobile and aerospace industries [2]. With that comes the need to be able to join metals to polymers. Such an assembly would produce a part that combines both the lightweight property of polymers with the properties of metals, such as strength and ductility, all at once. To further explore metal-to-polymer joining, Section 1.1 will present the different metal–polymer joining techniques that have been discussed in the literature. Later on, Section 1.2 will introduce friction stir welding (FSW) in the context of metal-to-polymer joining.

### 1.1. Metal-to-Polymer Joining Techniques

As mentioned earlier, plastics are joined with metals to form hybrid, strong yet light structures. At present, there are several methods used to join plastics to metals, such as adhesive bonding, mechanical fastening, and direct joining techniques. This section will briefly present the aforementioned metal-to-polymer joining techniques and will highlight the pros and cons of each method.

Firstly, adhesive bonding is a traditional joining method that uses a polymeric adhesive material between the joining pieces to create a joint. The strength of the joint mainly depends on the intermolecular forces between the joining pieces and the adhesive material. Thus, surface treatment for the pieces pre-bonding is crucial to enhance the joint durability and strength. Adhesive bonding is used in vast applications due to its ability to offer lightweight joints along with uniform stress distribution when subjected to load [3]. Many researchers investigated adhesive joint strength [4,5,6,7,8]. Yet, adhesive bonds have many limitations. Among these limitations are their sensitivity to environmental factors, especially temperature and humidity. Additionally, the bonded joints formed tend to fail instantaneously rather than degrade with time [3].

Another simple joining technique is mechanical fasting. While there are many different processes and members used in mechanical fastening, research is focused on riveting, as it has proven to produce reliable joints for metal-to-polymer joining [9,10,11,12]. The typical case of riveting is placing the metallic part at the bottom because it is exposed to the highest deformation while the polymer is placed on top of the metal; a rivet is punched through the polymer into the metal creating the joint. However, the main limitations of this process are the increased structure weight due to the use of an external rivet and the increased stress concentrations around the holes created for the rivets [3].

In recent decades, there has been a steady rise toward the research and development of alternative joining techniques in response to the growing demand for metal–polymer hybrid structures in industry and the limitations of conventional joining methods. For instance, ultrasonic welding (USW) is a process by which high-frequency, low-amplitude waves, typically in the range of 10–250 µm, are applied to the joint area while in the solid state to break the oxide layer and create the joint [13,14,15]. USW stands out for its low cost and short process time. However, for metal–polymer bonding, the difference in material performance is the main cause of USW formed joint high sensitivity to the applied waves vibrations amplitude which deteriorates joint strength [15]. Another metal–polymer welding process is laser welding, in which the joint is subjected to a laser beam, initiating bubbles of the plastic part to spread and diffuse in the molten solid interface forming the bond between the metal and the polymer. Laser welding produces strong joints due to the chemical bond between oxide film and carbon atoms, as well as mechanical bonds created as a result of van der Waals forces. The main drawbacks of laser welding are the utilization of shielding gas and sophisticated processes [3,16]. Injection molded joining is another metal–polymer joining process that injects the polymer into the mold through a nozzle. After the mold is filled, a pressure is applied to press the melt and avoid shrinkage during solidification [17,18,19,20]. This process stands out for producing complex shapes at a high production rate. However, low joint strength and additive pre-treatment requirements are considered among the major disadvantages of injection molding [19]. Friction-assisted joining is another category of metal–polymer joining where a tool plunges the metallic part into the polymer surface and rotates at a prescribed speed for a specific time [21,22,23,24,25,26]. An example is friction lap welding (FLW), in which the joint is created by applying pressure and heat using a non-consumable cylindrical rotating tool. The metal sheet is placed on the polymer. The tool is pressed against the metal sheet to ensure the desired pressure and is then rotated along the welding direction [27]. The heat generated due to friction between the metal and the tool melts the area of the plastic sheet adjacent to the metal creating the joint with the pressed metal. FLW produces strong joints. However, it is limited to creating overlapping joints in addition to non-uniform heat distribution across the weld line [27]. Table 1 summarizes the advantages and disadvantages for each method. However, because of the complexity of the machinery and the high production costs of the methods proposed thus far, new revolutionary joining methods are still required.

### 1.2. Friction Stir Welding as a Promising Metal-to-Polymer Joining Method

Industries and researchers alike are investigating new environmentally friendly methods for joining polymers to metals that produce strong joints while satisfying the market demand of low cost and energy. A new promising method is friction stir welding (FSW). FSW is one of the most recently developed welding processes. It attracts the interest of the scientific and the industrial communities alike [28]. It is defined as a solid-state joining process that uses a non-consumable rotating tool to join two adjacent parts. The tool consists of two parts: shoulder and pin. The pin is immersed into the abutting faces of the clamped parts until the shoulder touches the surface of the parts. Afterwards, the tool rotates and moves in the welding direction at a prescribed speed. Simultaneously, frictional heat is generated softening the base material creating a joint along the weld direction [29].

The Welding Institute (TWI, UK) invented FSW in 1991. In its early stages, FSW was intended for welding aluminum alloys [30,31,32,33,34,35,36,37]. Nonetheless, FSW proved its effectiveness in joining a wide variety of metals such as steel [38,39,40,41,42,43], magnesium [44,45,46,47,48], and titanium [49,50,51,52]. Successful joints were made of polymers such as Polyethylene (PE) [53,54], Polycarbonate (PC) [55,56], and Polymethyl Methacrylate (PMMA) [57,58]. Furthermore, the FSW process was utilized to join dissimilar metals [59,60,61,62,63,64,65,66,67,68], dissimilar polymers [69,70,71], and hybrid metal–polymer joints [72,73,74,75,76,77,78,79,80,81,82,83,84,85,86,87,88]. Therefore, the FSW process gained global recognition due to its potential in various applications in the automotive, aerospace, shipbuilding, and railway industries [89]. FSW’s main advantage is that it produces strong joints with fine microstructure, low shrinkage, and no cracks. Furthermore, FSW is an environmentally friendly process as it does not produce fumes or shielding gas and does not require consumables. It also consumes less energy and costs less to maintain compared to other similar joining processes [33,90]. Table 2 compares the process characteristics for the mentioned welding methods.

With the recent growing need of lightweight joints, several researchers have focused on the potential of the FSW process to produce dissimilar joints. Sahu et al. [62], Zhao et al. [66], and Derazkola [75] showed in their studies that rotational and traverse speed significantly affect the microstructures and mechanical properties of several dissimilar friction stir lap welding joints. Derazkola et al. tested the effect of the tilt angle and plunge depth on the joint strength. It was concluded that increasing the tilt angle in the range of 0–2°, while setting the plunge depth variation in the range of 0.1–0.4 mm, results in an optimum effect [88]. Ke et al. studied the thermal process of friction stir spot welding of dissimilar metals and provided a computational fluid dynamics model simulating the material flow throughout the process stages [65].

Several studies have focused on identifying the impact of the different types of tool shoulders and pin profiles on the microstructure and the mechanical properties of the joint. Mojtaba et al. showed that the weld quality is highly affected by the tool pin shape, unlike the welding process condition which is independent of the pin shape [69]. A similar conclusion was drawn by Kumar et al. when different shapes, sizes and materials of the tool pin and shoulder were reviewed [91].

Researchers have studied several properties of the weld joint. Microstructural investigations revealed that the stir zone consists of fine-grain structures that were created due to dynamic recrystallization [61,64]. Several studies reported a high joint strength for dissimilar materials using the FSW technique. Geng et al. achieved a joint strength of 75% of Al5052 when using the FSW process to join Al5052 to DP590 steel [63]. In a similar study, Saravana et al. joined Al6061 to Ti-6Al-4V and produced a joint with a strength of 87% of Al6061 [67].

**Figure 1:**
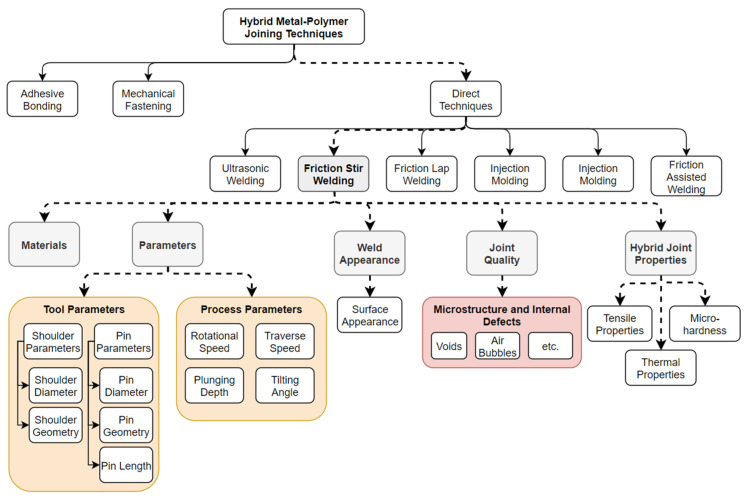
A summary of the topics reviewed in this work. The topics that are preceded by solid arrows are briefly reviewed, while the topics that are preceded with dashed arrows are discussed and reviewed in detail in the following sections of this work.

Over the past decade, researchers have been aiming to establish hybrid metal-to-polymer joints using FSW. Several promising joint strengths were reported in the literature. Dalwadi et al. reported a hybrid relative joint strength of 20% of PMMA welded between PMMA and AA6061 using FSW [79]. In a similar study, Patel et al. joined PC and AA6061 using FSW with a joint strength of 34% of PC [78]. Khodabakhshi et al. produced a dissimilar FSW joint with a joint efficiency of 50% relative to high-density polyethylene (HDPE) while joining HDPE and AA5059 [73]. Moreover, Haghshenas and Khodabakhshi provided a review paper addressing dissimilar joining of aluminum and polymer using FSW in terms of joint quality, soundness, material mixing, interfacial bonding, flow pattern, microstructure, mechanical properties, and thermo-mechanical modeling [92]. However, there is still a need for further investigations to understand the joining of metals to polymers using FSW. It is important, therefore, to summarize and discuss the findings of these works as a means of providing a reference to researchers of this field to facilitate further research on the utilization of FSW in joining metals to polymers. Thus, this work presents a comprehensive technical review on the FSW technique for joining metals to polymers by reviewing the reported literature. In addition, this work reviews and presents the latest practices reported in the literature aimed at enhancing the joint quality. Figure 1 presents the topics covered in this review paper.

To collect the data presented in this work, a systematic literature review was conducted through the SCOPUS database. The search keywords used were (“Friction Stir Welding” AND “Metal” AND “Polymer”). By searching for these keywords, 137 results were obtained. All papers with technical aspects related to this work were included. To be specific, works that reported findings on the impact of materials, tools, process parameters, and defects on the strength and microstructure of the produced joint were included. Other works that focused on other non-technical aspects were excluded.

The next section of this paper reviews the materials utilized in FSW, followed by a section reviewing the tools and process parameters. Thereafter, the joint quality and defects are discussed, and the joint mechanical and thermal properties are reviewed. Finally, the review paper is concluded with a summary, future outlook, and research gaps.

## 2. Materials

Aluminum alloys are the most utilized materials in FSW. However, other non-ferrous and ferrous alloys have also been investigated. Several researchers have been investigating ways of joining metals to polymers. Yet, this has been challenging due to numerous reasons. Firstly, metallic and polymeric materials have widely different surface energies, which has an impact on adhesive bonding at their interfaces. Secondly, metals and polymers have distinct structures: metals have crystalline structures with very high cohesive energy, whereas polymers contain long molecules of covalently bound carbon atoms with weak secondary forces between them. Furthermore, metals contained in polymers tend to form circular clusters rather than mixing, resulting in limited metal solubility in polymers. The deterioration of polymers is particularly critical in this case, especially when metal–polymer joining is achieved via welding. The major source of deterioration is the metals’ high hot-working temperatures, which are often higher than polymer decomposition temperatures. This can lead to oxidation, molecular weight loss, or polymeric molecule fracture [73]. Therefore, to join the work pieces successfully, it is important to choose the materials based on their thermal properties, where the difference between the metals’ hot working temperature range and the polymers’ decomposition onset temperature is minimal. Different joint configurations have been reported in the literature, as presented in Table 3. In a butt joint, the work pieces are placed adjacent to one another with the metal on the advancing side (AS) (with tool rotating direction). This is mainly because the harder material is more convenient to transport compared to the softer material when positioned in AS [77]. Likewise, the metal is placed on the upper side of a lap joint.

## 3. Tools and Process Parameters

### 3.1. Tool Parameters

The most important part of the FSW process development is the tool geometry. Material flow is governed by the tool geometry, which, in turn, governs the traverse rate at which FSW may be performed [93]. The primary geometry features of FSW tools are the shoulder diameter and feature, the pin diameter and feature, and the pin shape and length [94]. The tool is mainly fabricated from steel and serves two main purposes: localized heating and material flow. The primary cause of the tool plunge heating up in early stages is the friction between the pin and the workpiece. In addition, deformation of the material causes some extra heating [93]. The tool is plunged into the workpiece until the shoulder of the tool contacts the workpiece surface. The most significant source of heat is friction between the shoulder and the workpiece. The shoulder also accommodates the volume of the heated material. The standard type of shoulder design is the concave shoulder. The simple, easily machined concavity design is fabricated by a small angle between the edge of the shoulder and the pin. This shoulder type needs the tool to be tilted by 2° to 4° from the normal of the welding line away from the direction of weld travel [95]. Shoulders may contain features that can be machined onto any tool shoulder profile. These features increase the amount of material flow and lead deformed material from the edge of the shoulder to the pin, thus eliminating tool tilting requirement [95]. Scrolled and grooved are reported shoulder features in the literature used to enhance the weld quality in hybrid metal–polymer joints [83,86]. A large shoulder diameter increases the frictional heat input, which enhances material flow, obtaining high quality joints. On the contrary, a small shoulder diameter results in an insufficient heat input and material flow, leading to a defect at the stir zone (SZ) [94]. Therefore, the size of the pin and shoulder is crucial in terms of heating.

Moreover, tool pin geometry affects the weld appearance and determines the consistency of the microstructure which are important characteristics of a high-quality weld [96]. The most common used pin geometries to successfully join metals to polymers and obtain relatively strong welds were cylindrical threaded [72,73,79,83,86] and tapered pin geometries [74,75,76,77,78,85,87]. However, Huang et al. concluded that using a tapered thread pin with triple facets can produce more pulsating action. Thus, the material transfer is improved compared to a thread-tapered pin and a weld strength of 20.6% of the polymer’s strength is achieved using the friction stir lap welding (FSLW) technique [82]. The pin length and diameter likewise affect the weld appearance. Large pin dimensions may fail to induce the plastic flow, creating a rough surface and large valley-like defects at the joint area [77]. The welding tools that have been used in the literature during conventional FSW of metals to polymers are listed in Table 4.

### 3.2. Process Parameters

The FSW process depends on four main parameters: tool rotational speed, traverse welding speed, plunge depth, and tilt angle. Each of these parameters are independent, though each has a significant effect on the joint quality and weld appearance.

#### 3.2.1. Plunge Depth

By rotating, tilting, and plunging the permanent probe on the material’s surface, the softened and plasticized material is stirred and moved away from the advancing side towards the retreating side (RS) and solidifies under the forging force of the probe [72]. Several published papers recorded that a defect-free joint is obtained at low (usually 0.2–0.5 mm) plunge depths; by increasing the plunge depth, the area of the SZ decreases [72,73,74,75,76,77,78,82,85]. At high depth rates, flashes may form on the material surface due to the excessive axial forces moving the material away from the welding line [74].

#### 3.2.2. Tilt Angle

The tilt angle affects the material flow of the weld. A low tilt angle may lead to tunnel- and crack-like defects in the welds [96]. When increasing the tilt angle, the forging force to plasticize the polymer increases and fills the defects better while bonding with metal fragments [74,87,96]. However, by increasing the tilt angle, the area of the stir zone (SZ) decreases; thus, high tilt angles may cause material overflow from the SZ [75].

#### 3.2.3. Traverse Speed

The traverse speed contributes to the size of the metal fragments within the polymer. At high traverse speeds, both length and thickness of the metal fragments are small, and as the traverse speed decreases, the fragments sizes grow, resulting in a better mechanical interlocking [75,80]. This is because decreasing the traverse speed increases the preheat temperature and duration of the weld process, leading to a better material transfer and, hence, larger metal fragments [94].

#### 3.2.4. Rotational Speed

Likewise, increasing the rotational speed enhances the heat input; thus, longer and wider metal fragments are generated within the SZ [72]. In general, the sizes of the fragments in the advanced side of the material are larger than that at the retreating side due to the counterclockwise rotation of the tool. However, high rotational speeds cause defects, including micro-voids [72], wider gaps [85], and tunnels [73], which deteriorate the joint strength. On the other hand, low rotational speeds may not be adequate to stir the material due to low heat input in addition to the presence of wormhole defects because of the insufficient material flow [73,85]. Therefore, process parameters must be optimized to avoid defects and obtain high-quality welds. Table 5 shows optimum process parameters reported in the literature for the FSW process between metals and polymers.

## 4. Joint Quality and Defects

### 4.1. Surface Appearance

To better understand the role of the FSW process parameters on the joint between polymers and metals, assessing the weld appearance and examining the visual surfaces and cross-sections of the joint are required. Rahmat et al. studied the effect of plunge depth optimization on the joint quality. At a plunge depth of 0.1 mm, a valley-like structure was observed on the joint surface. However, by slightly increasing the depth to 0.2 mm, a defect-free joint was created, increasing the value further leading to the presence of residual material on the advancing side (AS) [77].

Gao et al. [80] and Shahmiri et al. [85] obtained rougher joint surfaces by increasing the tool rotational speed and lowering the traverse speed, respectively, for different materials. This is because such a combination increases the thermal input, which causes larger metal fragments to mix with the polymer, producing a rougher surface. Nonetheless, operating at overly high rotation speeds causes the polymer to overflow due to its large thermal expansion [87]. Huang et al. reported a case of overflow in an attempt to join AA6061 to PEEK by the FSLW process. It was observed that the overflow gradually decreases as the traverse speed increases [82].

**Figure 2:**
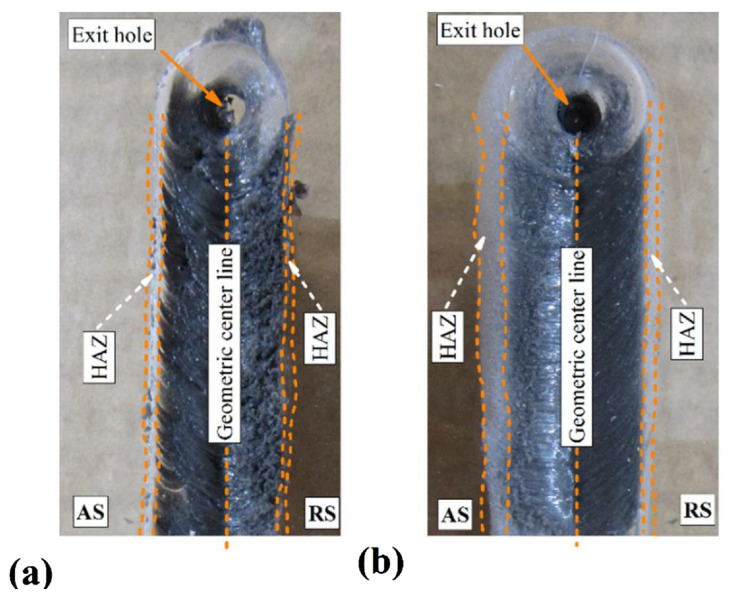
Surface material flow of joint at (**a**) 1940 rpm/45 mm min^−1^ and (**b**) 960 rpm/90 mm min^−1^ Reprinted with permission from Ref. [75]. 2018, H.A. Derazkola, M. Elyasi.

Contrary to common practice, Derazkola and Elyasi [58] and Derazkola et al. [88] performed the FSLW process while placing the polymer at the top, facing the tool probe, and the metal at the bottom to increase the cooling rate after removing the tool. Both studies observed the presence of grey joint lines at different process parameters, as illustrated in Figure 2. The gray color indicates the mixing of the metal alloy and the polymer sheet at the SZ. The weld appearance and material overflow were smooth.

### 4.2. Microstructure and Internal Defects

**Figure 3:**
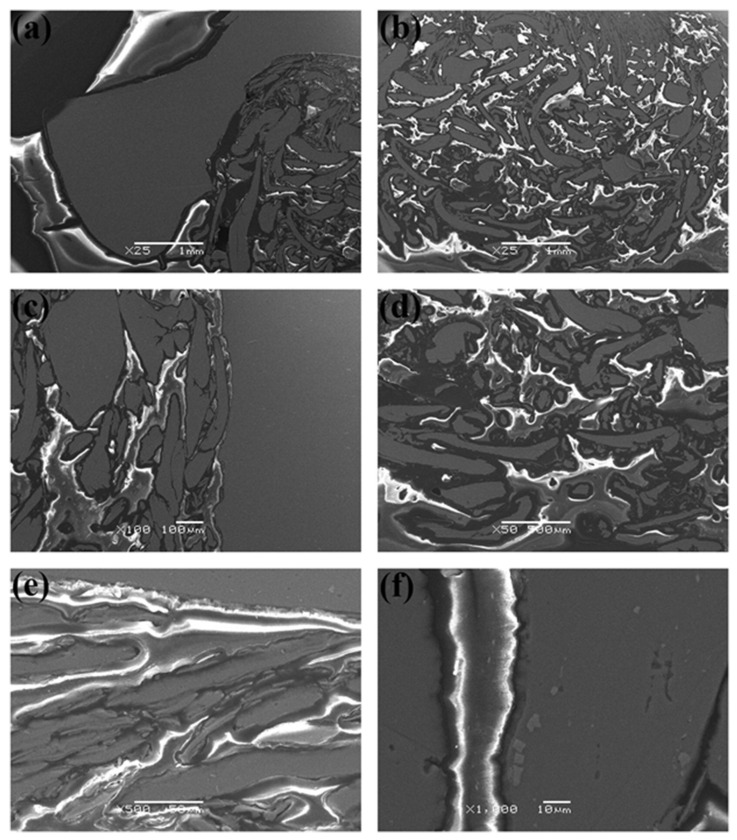
SEM images for the aluminum-polymer interfaces in the intermixed regions of the stir zone; where bright gray contrast is Aluminum layers, dark gray matrix is melted and re-solidified polymer, dark spots through polymer matrix are shrinkage voids, and mixed gray–white contrast at interface presents the solid state modified polymer via FSW. Reprinted with permission from Ref. [73]. 2014, F. Khodabakhshi et al.

An excellent joint surface appearance may be obtained using different parameters. However, the performance and strength of the joint vary due to the joint’s microstructural difference in the SZ. One of the most common techniques used to observe the microstructure is scanning electron microscopy (SEM). The dominant joining mechanism is mechanical interlocking between the metal and the polymer at the SZ [73,75,79,80]. The severely deformed metal penetrates the softened polymer and leads to the formation of metal interlocked fragments. In general, metal fragments improve the mechanical performance of the joint. Figure 3 illustrates the mechanical interlocking between the metal and the polymer matrix, where the bright gray contrast resembles the metal, and the dark gray continuous matrix is the melted and resolidified polymer [73].

**Figure 4:**
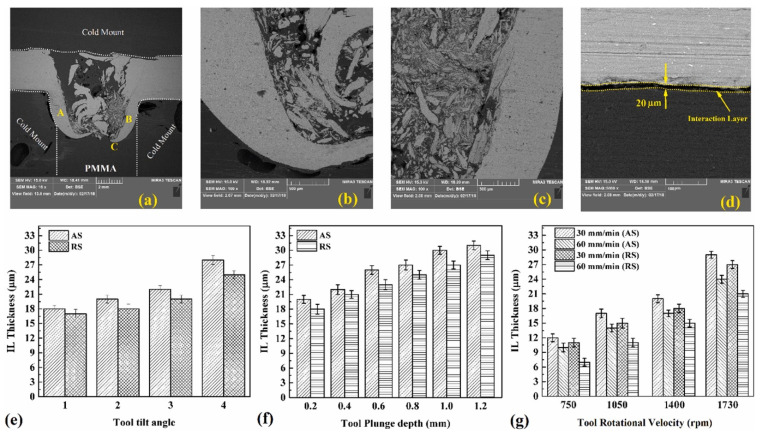
Magnification SEM image of the (**a**) the showing side, (**b**) the retreating side (Label A), (**c**) the advancing side (Label B), and (**d**) the interaction layer (Label C). Effects of the (**e**) tool tilt angle, (**f**) tool plunge depth, and (**g**) tool rotational velocity on the thickness of the interaction layer. Reprinted with permission from Ref. [74]. 2019, H.A. Derazkola, A. Simchi.

Heat input affects the size of the interaction layer that contributes to the adhesion bonding between the metal and the polymer. Derazkola and Simchi studied the effect of the process parameters on the thickness of the interaction layer in both advancing and retreating sides [74]. Aluminum fragments emerge and spread in the contact layer. Smaller aluminum fragments are distributed in the RS (labeled as “A”) compared to the AS (labeled as “B”). Furthermore, uniform flow patterns, such as onion rings, tunnels, and kissing boundaries, are not observed. During the FSW process, low-temperature severe plastic deformation results in the creation of aluminum pieces in the SZ, which strengthen the joint area. Stretched aluminum ramus creates a wavy boundary at the Al–polymer contact, as shown in Figure 4c. This wavy border line functions as a mechanical interlock between the aluminum alloy and the polymer sheet. Figure 4d shows a magnified image of the contact between the aluminum ramus and the polymer matrix (labeled as “C”). It appears that a contact layer forms between the outside portion of the aluminum ramus and the polymer. The interaction layer thickness is larger at the AS due to a greater heat input. Likewise, the thickness increases with incrementing process parameters such as rotational velocity, tilt angle, and plunge depth, as shown in Figure 4e,f. However, as discussed in the previous section, an excessive generation of heat input (a high rotation speed and/or a low traverse speed) increases the amount and size of metal fragments at the SZ, and the more metal fragments, the less the contribution from the polymer towards the joint formation, leading to less mechanical interlocking, thus decrementing the joint strength [74].

**Figure 5:**
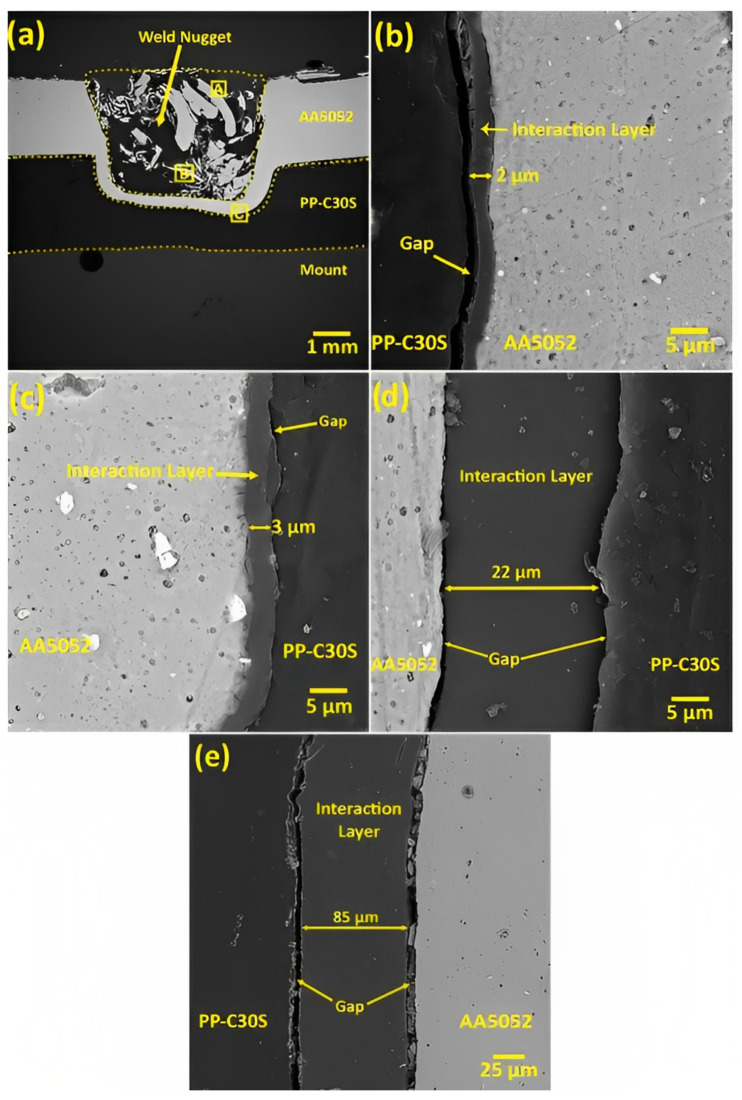
FE-SEM images of the metal/polymer interface at different regions: (**a**) cross-section and regions selected for investigation in the 800-70 specimen; (**b**) interaction layer at “region A”; (**c**) interaction layer at “region B”; (**d**) interaction layer at “region C”; and (**e**) interface of the polymer sheet and the weld nugget for the 1200-70 specimen. Reprinted with permission from Ref. [85]. 2016, Shahmiri et al.

Shahmiri et al. reported the formation of a gap between the interaction layer and the polymer matrix in the stir zone [85]. The gap was formed due to the significant differences between the metal’s and the polymer’s coefficients of thermal expansion. During the cooling stage, polymers shrink faster than metals, leaving a gap at the interaction layer, as shown in Figure 5b–e. By comparing the size of the interaction layer in Figure 5d,e, which were formed at rotation speeds of 800 and 1200 rpm, respectively, it can be noticed that the thickness of the interaction layer in Figure 5e is larger. This confirms the previous results obtained in [74]. However, the gap formed in Figure 5e is wider than that of Figure 5d; thus, the gap width increases with more generated heat input. This showed that the chemical adhesive bonding at the interaction layer between the polymer and the metal is weak and the mechanical interlocking is the dominant bond.

**Figure 6:**
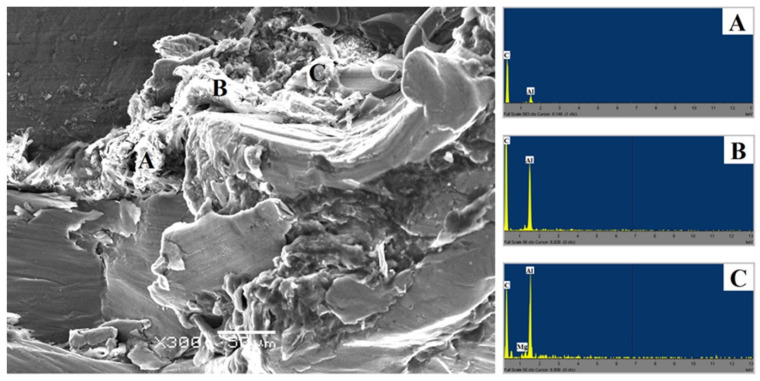
FE-SEM image with related EDS spectra from the surface of transverse tensile dissimilar Al–HDPE FSW failed jointat HDPE matrix (point **A**), and AA5059–HDPE interface (points **B** and **C**). Reprinted with permission from Ref. [73]. 2014, F. Khodabakhshi et al.The most frequently reported internal defects in the literature are voids [73,75,76,77,80]. Voids appear as dark spots in the polymer matrix, as shown in Figure 7. The main cause of void formation is the incompatible thermal deformation of dissimilar materials [87]. Polymers lose their molecular weight as a result of melting and re-solidification. This phenomenon intensifies their shrinkage properties, creating voids [75].

Energy dispersive spectroscopy (EDS) examinations of the white contrast areas in Figure 6B,C show the presence of carbon on the polymer side. As a result, these regions may have some polymer attached to the aluminum alloy, providing additional evidence for bonding at the aluminum–polymer contact during the FSW process. This adds credibility to the theory that the bonding was accommodated by a connection between aluminum oxide and the polymer, which may have been aided by mechanical interlocking via polymer penetration into nano-scale holes, and perhaps molecular bonding, which is a mixed regime of Al–O–C–Mg components. It also showed that aluminum and magnesium oxidation occurred, which may have contributed to the strength of the created contact between the polymer and the metal [73]. Therefore, it is discovered that the FSW parameters had an impact on the chemical composition of the contact zone. At larger heat inputs, more oxygen is present in the interaction layer, indicating a more extensive breakdown of the polymer and in situ reactivity with the base metal to generate oxides. It is also concluded that any linking between the polymer and the surface aluminum oxide is either secondary or van der Waals bonding [74].

Bubbles and pores are also common defects found at the interface between the metal and the polymer. Their generation indicates the presence of structural water and/or trapped air in the polymer [82]. During the FSW process, the melted polymer absorbs humid air from the surroundings. Due to high temperature, the moisture expands as it turns to vapor, creating the bubbles [75]. Although internal defects deteriorate the joint strength, it must be noted that the causes of the mentioned defects are not yet conclusive. However, internal defects may be evaded by optimizing the heat input in the SZ area, choosing the right weld tools, and operating at reasonable process parameters, resulting in a defect-free joint with decent mechanical performance [74,75].

**Figure 8:**
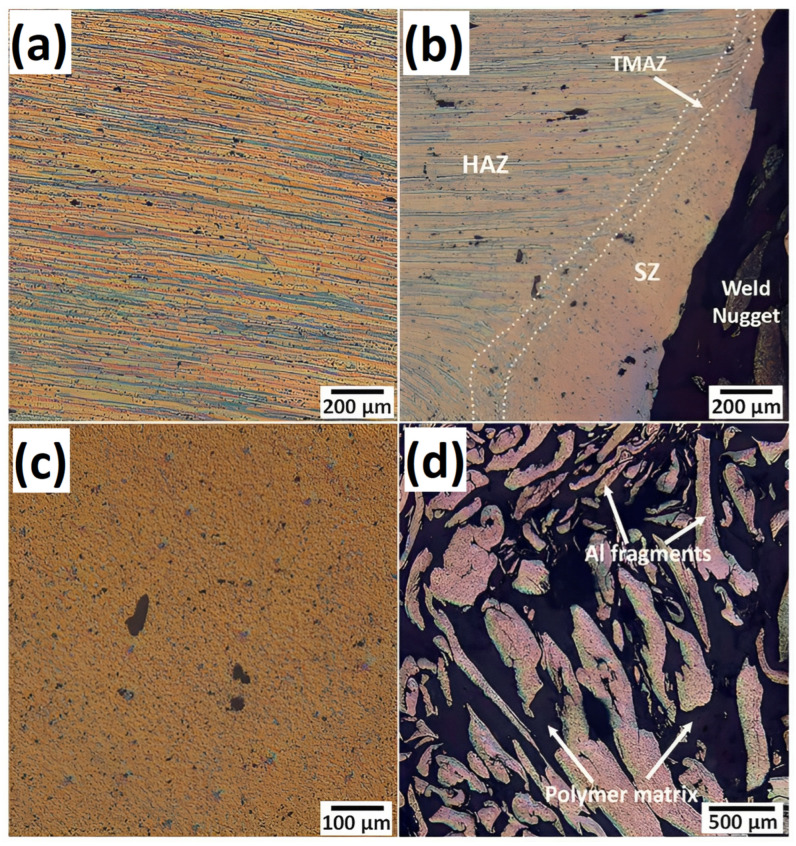
Microstructure of different regions of the metal/polymer structure: (**a**) base metal; (**b**) area near the weld nugget; (**c**) magnified SZ in (**b**) and (**d**) fragments of the metal inside the polymer matrix at the weld nugget. Reprinted with permission from Ref. [85]. 2016, Shahmiri et al.

**Figure 9:**
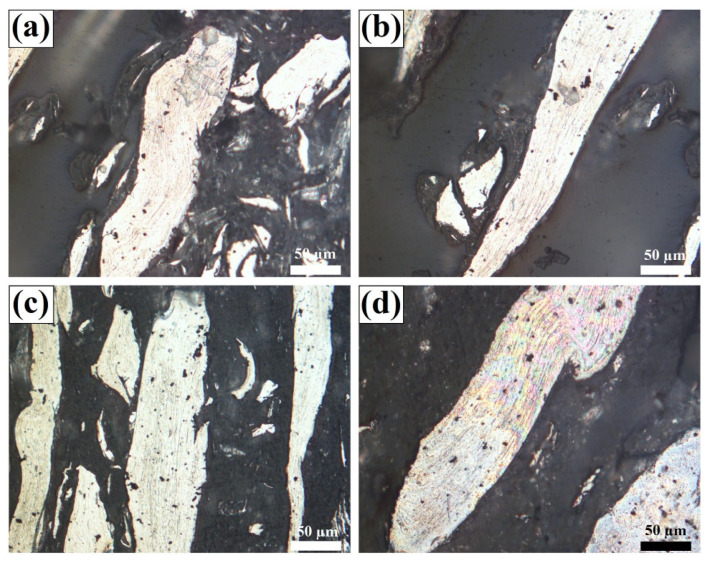
Effects of processing parameters on the grain structure of metal fragments in the polymer matrix joint: (**a**) *w* = 800 rpm-*v* = 30 mm/min, (**b**) *w* = 800 rpm-*v* = 50 mm/min, (**c**) *w* = 1000 rpm-*v* = 30 mm/min, and (**d**) *w* = 1000 rpm-*v* = 50 mm/min. Reprinted with permission from Ref. [72]. 2021, S.M. MirHashemi et al.

Under the thermal cycle and mechanical stirring of the FSW tool, it is important to analyze the grain structure and the changes in properties of the metal. Shahmiri et al. observed the grain structure of the joint at different areas, as shown in Figure 8 [85]. The base metal has an elongated grain structure due to the rolling manufacturing process. The grain structure remains unaffected in the HAZ. In the TMAZ, only partial recrystallization occurs, as the temperature is not adequate to induce dynamic recrystallization. Moreover, the grains were deformed and extended towards the direction of stirring action of the rotating pin. In the SZ, the metal was exposed to high strain and elevated temperatures. As it can be seen in Figure 8c, the extended grains were renovated into very fine equiaxed grains in the SZ because of dynamic recovery/recrystallization. Similar results were achieved by Huang et al., who reported the microstructure of the aluminum anchor in the SZ to be equiaxed, with an average grain size of approximately 35 μm [82]. MirHashemi et al. studied the effects of rotational and traverse speeds on the grain structure of aluminum fragments inside the SZ for dissimilar LDPE-AA7075 weldment, as illustrated in Figure 9 [72]. As it can be found, by increasing the rotation speed and/or decreasing the traverse speed, the mixing action of the FSW treatment was increased, which led to the formation of more aluminum fragments inside the polymer matrix with a more refined and recrystallized grain structure.

## 5. Hybrid Joint Properties

### 5.1. Tensile Properties

Various studies were aimed at understanding the effects of different process parameter variations on the joint strength [74,75,76,82,83,85,86]. Table 6 illustrates the tensile strength for metal–polymer hybrid joints at different process parameters obtained from the literature [78]. The highest tensile strength recorded was at 14.9 MPa for specimen E at 500 rpm and 40 mm/min. By comparing specimen E with K, it is observed that a slight change in the feed rate severely deteriorated the tensile strength. A similar observation is made for the rotational speed by comparing specimens E and T. Hence, it can be concluded that FSW is a parameter-sensitive technique. Various papers in the literature concluded that the joint strength increases with rotational speed and then decreases after reaching a certain point [75,78,79].

The tensile strength deteriorates at high rotational speeds because of the excessive heat input from friction between the material and the tool, which results in defects, as discussed in previous sections. Likewise, the tensile strength increases to reach a peak and then decreases as the traverse speed increases [73,85]. Additionally, the traverse speeds are usually set at relatively low rates (30–100 mm min^−1^) to provide sufficient time for stirring. When increasing the tilt angle within the range of 0° to 2°, the intermixing of dissimilar materials was improved. The tool plunge depth optimization in the range of 0.1 to 0.4 mm possessed an optimal effect on the joint strength [88]. However, increasing the tool plunge depth and tilt angle beyond such ranges forces more material to be ejected from the SZ. This is due to the hefty heat generated that causes material overflow and surface defects, such as flashes, leading to a lower joint strength [74]. Moreover, increasing the tilt angle along with decreasing the plunge depth leads to intensifying air bubble formation within the solidified polymer. This can depreciate the mixed zone characteristics and quality [88].

To enhance the joint quality, eliminate weld defects, and increase weld strength, several researchers introduced new procedures to the FSW process for welding dissimilar materials. For example, Wang et al. introduced groove micro-textures through a laser ablation pre-treatment on the aluminum alloy surface prior to joining with PA6 using FSW [81]. For joints with V-shaped grooved aluminum plates, the bonding mechanism was attributed to the large mechanical interlocking from the geometric grooves in the micro-textures and the development of a C–O–Al bond at the interfaces. Upon fracture, the PA6 plate underwent severe plastic deformation and subsequently fractured with a maximum shear-tensile load of 1194 N, which exceeded the yield strength of the PA6 base material with a cross-sectional area of 20 × 1.5 mm^2^. MirHashemi et al. placed silicon carbide (SiC) nanoparticles on the dissimilar FSW of low-density polyethylene (LDPE) and AA7075 aluminum [72]. The primary purpose was strengthening the polymer side during the joining process by pre-placing the particle in the polymer side which enhanced the dissimilar intermixing process and the material flow profile. The dissimilar joining strength ratio was significantly enhanced up to around 70% of the LDPE strength.

**Figure 10:**
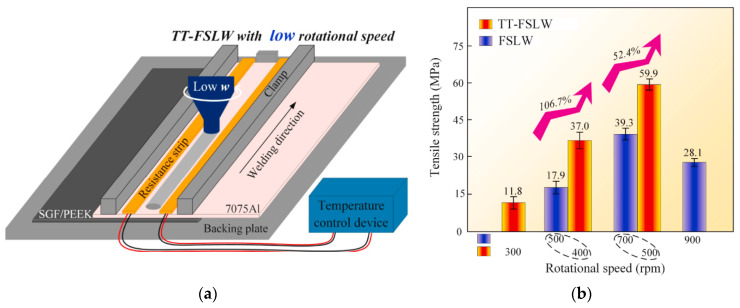
(**a**) Schematic diagram of the TT-FSLW process; (**b**) effect of rotation velocity on tensile shear strengths of joints under different processes. Reprinted with permission from Ref. [87]. 2021, M. Li et al.

A similar approach was applied by Derazkola and Simchi, in which alumina nanoparticles were fed in the joining line during the process of obtaining AA6062/PMMA joints. However, there was no need to preplace the particles at the welding interface by machining a cavity. The process is introduced as friction fed stir welding (FFSW). The strength of the joint using conventional FSW was around 49 MPa, which is 64% of PMMA’s tensile strength. The addition of Al_2_O_3_ nanoparticles increased the tensile strength to 61 MPa (∼30% improvement) [76]. Li et al. developed a new technique named top thermic friction stir lap welding (TT-FSLW) [87]. Based on thermal tensioning, two heating strips of different resistance are set up in parallel on both sides of the weld (see Figure 10a) to increase the compressive plastic strain during the FSLW process. This is to minimize the stress during the process and residual stress after welding. Figure 10b illustrates the TT-FSLW joint strength at 400 and 500 rpm, which are 37.0 MPa and 59.9 MPa, respectively. These values present an increase of 106.7% and 52.4% compared with 17.9 MPa and 39.3 MPa of the FSLW joint at 500 and 700 rpm, respectively. Moreover, the effect of rotation velocity is obvious, which increases to reach a peak at 39.3 MPa for FSLW, then decreases at higher speeds, which agrees with the results obtained from the literature [75,78,79].

### 5.2. Microhardness

**Figure 11:**
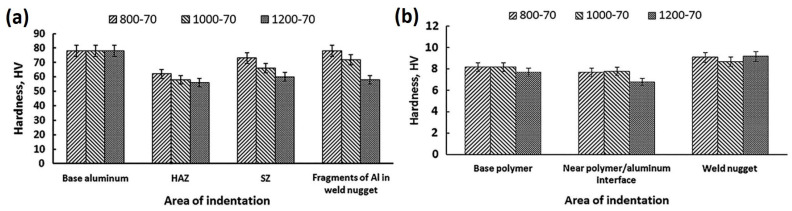
Rotational velocity effect on weld zones hardness at the (**a**) metal and (**b**) polymer. Reprinted with permission from Ref. [85]. 2016, Shahmiri et al.

**Figure 12:**
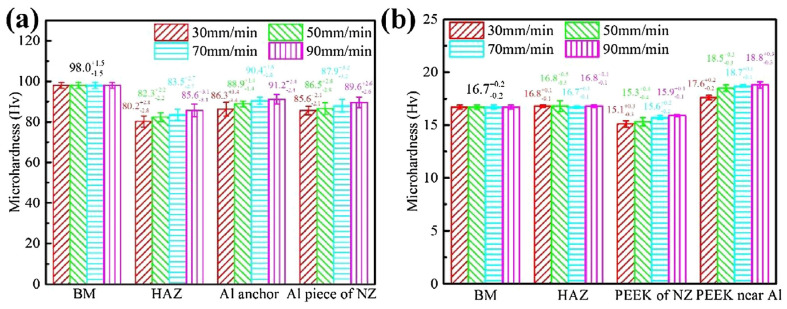
Traverse speed effect on weld zones hardness at the (**a**) metal and (**b**) polymer. Reprinted with permission from Ref. [82]. 2018, Y. Huang et al.

In order to test the hardness of the welded joints, the hybrid joints are indented at different regions. On the polymer side, researchers reported the lowest hardness value obtained for melted–resolidified polymers at the SZ area [72,73,75,82,85], presumably due to the loss of molecular weight of the polymer and the reduction of crystallinity. However, the average hardness value of the SZ is greater than that of the base polymer because of the existence of embedded metal fragments at the metal–polymer interface [73,82]. The metal pieces had a fine-grain structure with high hardness, as they were exposed to high strains before being cut using the FSW tool. Thus, process parameters such as tool rotational and traverse speeds, that contribute to the size and number of metal pieces at the SZ will impact the overall hardness [74]. On the metal side of the joint, the hardness decreases at the SZ, TMAZ, and HAZ areas due to the recovery process [85]. During the FSW process, the metal softens and loses its pre-cold-worked strength after recrystallization, due to the applied heat input during joining. Moreover, the hardness at the SZ is higher than that at the HAZ, due to the small size of grains at the SZ resulting from the Hall–Petch effect [85]. Huang et al. and Shahmiri et al. measured the microhardness for different zone areas using the Vickers test [82,85]. Both studies recorded the minimum hardness at the HAZ on the metal side. This reduction in hardness was due to the decrease in dislocation density and the growth of sub-grains during the recovery process, as well as partial recrystallization. However, on the polymer side, the HAZ hardness remained unchanged. This is due to the polymer’s low thermal conductivity. Moreover, increasing the tool rotational speed reduced the weld hardness, regardless of the zone area, as seen in Figure 11. On the contrary, Figure 12 shows that increasing the traverse speed increases the weld hardness. The hardness value of the metal fragments increases with the increase in the welding speed, which may be advantageous to the tensile bond properties of the hybrid joint [82]. In general, the effect of the process parameters on hybrid metal-to-polymer joint hardness is inversely proportional. High heat input (high rotational speed, tilt angle, and plunge depth) softens the metal and decomposes the polymer.

### 5.3. Thermal Studies

**Figure 13:**
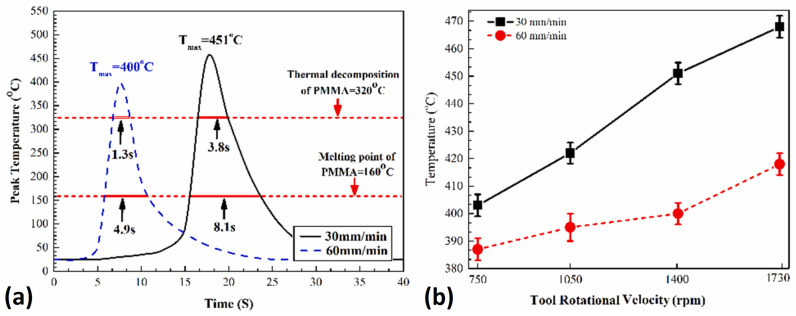
(**a**) Temperature variation for the aluminium/PMMA joint; (**b**) relation between tool rotational velocity and peak temperature. Reprinted with permission from Ref. [74]. 2019, H.A. Derazkola, A. Simchi.

Experimental and simulation results have shown that friction produces more than 90% of the heat generated during the FSW process [76]. The welding temperature in the FSW process affects the material flow and the welding stress [87]. Thus, all FSW parameters that influence the heat input to weld the specimens should be optimized to provide the necessary heat input. Thus, it is important to examine the thermal profile of the joint forming. Figure 13a shows the trend of the joint area temperature throughout the process. The temperature of the joint area increases rapidly to reach a peak above the polymer’s decomposing temperature for a short period of time. Subsequently, the cooling stage begins at a slow rate due to the low heat transfer coefficient of the polymer; hence, the temperature fields remain high at the weld line [75]. The peak temperature always exceeds the melting point and may reach a higher value than the thermal decomposition point of the polymer, resulting in local chemical and physical changes to the material [74,75,76]. However, the peak temperature steady-state time is relatively short. Thus, complete polymer degradation is unlikely to occur.

**Figure 14:**
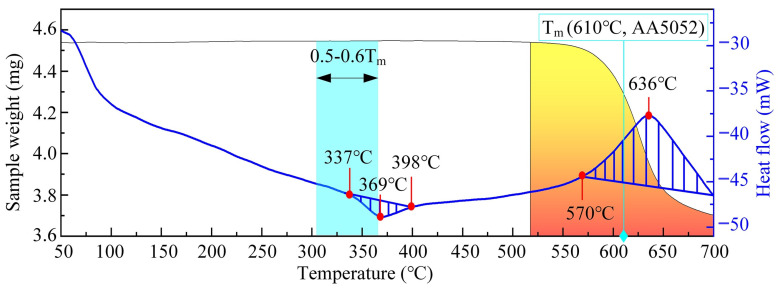
Thermal characteristics of CF-PEEK. Reprinted with permission from Ref [84]. 2021, H. Dong, Z. Tang, P. Li et al.

Moreover, increasing the traverse speed reduces the peak temperature of the FSW process and enhances the cooling rate that contributes to better joint properties. Figure 13b illustrates the change of peak temperature with rotational speed. Increasing the rotational speed generates more heat, resulting in higher elevated temperatures. Similar behavior was reported for other FSW process parameters, such as tilt angle and plunge depth [74]. Dong et al. analyzed the thermal characteristics of CF-PEEK, as illustrated in Figure 14 [84]. The sample weight of the PEEK remained constant until the thermal degradation point of 570 °C. Afterwards, the polymer lost a significant amount of its molecular weight, which deteriorated its properties. Moreover, the interfacial peak temperature of aluminum–polymer joints is estimated to be around 0.5–0.6 of the melting point of aluminum alloys. Therefore, the risk of thermal degradation may be eliminated by choosing materials that are thermally compatible, where around half the metal temperature is lower than the thermal decomposition temperature of the polymer.

**Figure 15:**
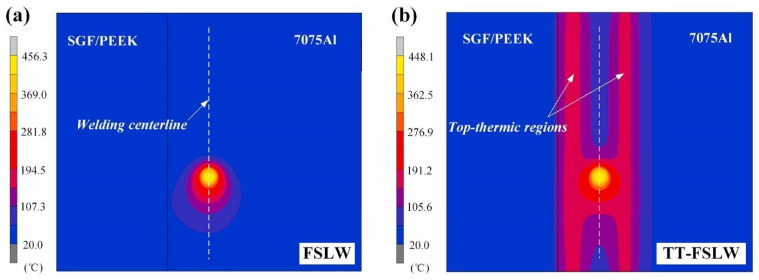
Temperature fields for (**a**) FSLW at 700 rpm and (**b**) TT-FSLW at 500 rpm. Reprinted with permission from Ref [87]. 2021, M. Li et al.

At present, the welding temperature at the interface is difficult to be obtained experimentally. Thus, a numerical simulation method may be used to predict the thermal profile of the joint. Li et al. simulated the temperature distribution for FSLW and TT-FSLW to understand the TT-FLSW process of the aluminum alloy and polymer hybrid weld, as shown in Figure 15 [87]. At 700 rpm for FSLW and 500 rpm for TT-FSLW, the center temperatures reached 457.1 and 448.6 °C, respectively. FSLW at high rotational speed and TT-FSLW at low rotational speed both produced about the same welding temperature. The high temperature zone had a H-shaped distribution, and the parallel top-thermic regions reduced the temperature gradient surrounding the welding heat source. During TT-FSLW, the weld center experienced both preheating and post-heating impacts. As a result, when compared to FSLW, TT-FSLW had lower heating and cooling rates. Meanwhile, the preheating and post-heating temperatures in the weld center were both below 200 °C due to the heat conduction distance between the resistance heating strips and the weld for TT-FSLW.

## 6. Summary

In this work, the friction stir welding (FSW) process for solid-state dissimilar joining of hybrid metal–polymer structures was reviewed and discussed. This was achieved by reviewing different works in the literature that have studied the effects of the different materials utilized in the process, the used tool types of the pin and shoulder profile, and the different process parameters on the joint’s quality, strength, hardness, microstructure, and thermal profile. It can be concluded from the review conducted that in order to obtain the best weld quality, the following points should be considered:

- Joining metals to polymers is challenging due to huge differences in their properties. However, choosing soft metals, such as aluminum and magnesium, along with high thermal decomposition polymers would successfully form a successful joint using wide range of processing parameters.
- A large shoulder diameter with a concave feature and a short, tapered pin enhances the material flow through the weld joint that directly improves the joint strength.
- Increasing the heat input through the weld joint enhances the strength. This can be achieved by increasing the tool rotational speed, tilt angle and plunge depth, or by decreasing the traverse speed. However, excessive heat input can cause polymer degradation and lead to internal defects decreasing the joint quality. Hence, it is critically important to optimize the process parameters to provide the optimum heat input.
- The hardness of the stir zone reduced as the heat input increased and varied among locations. The hardness was altered by thermal degradation of the polymer and variations in crystallization degree.
- Internal defects such as voids, bubbles, and pores, as well as the contact layer between the metal and polymer, were discovered to have a substantial impact on the mechanical strength of joints.
- Embedded metal pieces in a solidified polymer matrix are found in the microstructure of the FSW joint cross-section.
- The bonding mechanisms throughout the joining process are major mechanical interlocking via the creation of micro- and macro-constraints in addition to a minor interfacial chemical adhesion between the metal and consolidated polymeric layers.

## 7. Research Gaps and Future Outlook

FSW researchers are interested in metal–polymer hybrid structures. However, several study areas of FSW of metal-to-polymer joints must be addressed in order to better understand the joint properties and increase the utilization of FSW technology in joining metals to polymers. These areas include the following:

- In depth examination of the flow mechanism of deformed metal under the impact of stirring tools during welding, with the aim of increasing weld metal flowability and the service life of FSW tools.
- Research could be extended to better comprehend the principles of thermo-mechanical interactions and material flow characteristics during welding to produce strong joints by experimenting with different combinations of input parameters to increase the efficiency of the FSW of dissimilar materials.
- Extensive investigation of the binding between metals and polymers at the microscale is needed to manage and eliminate micro-flaws in order to increase joint strength and create joints with excellent overall properties.
- Evaluation of joint fatigue, bending, and toughness to better understand the feasibility of FSW hybrid joints in applications involving dynamic conditions.

## Figures and Tables

**Figure 7:**
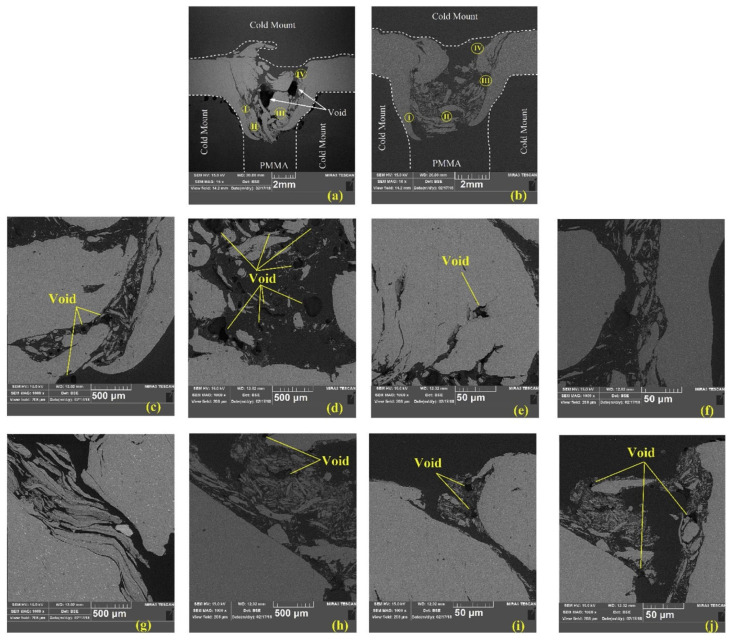
Cross-sectional SEM images of AA6063/PMMA joints. The samples were prepared by (**a**) FSW and; (**b**) FFSW; (**c**–**f**) shows the details of SZ regions (I-IV) in (**a**) respectfully; (**g**–**j**) shows the details of SZ regions (I-IV) in (**b**) respectfully. Reprinted with permission from Ref. [76]. 2020, H. Aghajani Derazkola and A. Simchi.

**Table 1:** Advantages and disadvantages of metal-to-polymer joining methods.

Method	Advantages	Disadvantages	References
Adhesive bonding	Uniform stress distribution	Sensitive to the environment	[3]
	Light weight structure	Sudden failure	
Riveting	Simple process	Increased structure weight	[3]
	Structure flexibility	Stress concentrations present	
Ultrasonic welding	Complex shapes produced	Usage of shielding gas	[15]
	High production rate	Presence of bubbles	
		Low joint strength	
Laser welding	Strong joints produced	Usage of shielding gas	[16]
		Presence of bubbles	
Injection molding	Complex shapes produced	Low joint strength	[19]
	High production rate		
Friction lap welding	High joint strength	Non-uniform heat distribution	[23,27]

**Table 2:** Process characteristics for metal–polymer welding methods.

Joining Characteristics	Injection Molding	Ultrasonic Welding	Laser Welding	Friction Lap Welding	Friction Stir Welding
Heat source	External (pre-melted polymer by furnace)	External (high-frequency ultrasonic waves)	External (laser beam)	Internal (instantaneously generated frictional heat)	Internal (instantaneously generated frictional heat)
Easily automated	No	Yes	Yes	Yes	Yes
Equipment cost	High	High	High	Low	Low
Environmentally friendly	No	No	No	Yes	Yes
Joining configuration	Limited to overlapping joints	Limited to overlapping joints	Limited to overlapping joints	Limited to overlapping joints	Various configurations (butt, lap, etc.)
Joint strength	Weak	Weak	Strong	Strong	Strong

**Table 3:** Hybrid metal-to-polymer joints reported in the literature.

Joint Configuration	Metal	Polymer	Reference
Butt joint	AA5059	High-density polyethylene (HDPE)	[73]
	AA6061	Polycarbonates (PC)	[78]
	AA6061	Poly (methyl methacrylate) (PMMA)	[79]
	AA7075	Low-density polyethylene (LDPE)	[72]
	AA7075	Polycarbonates (PC)	[77]
Lap joint	AA5052	polypropylene homopolymer (PP-C30S)	[85]
	AA5058	Polycarbonates (PC)	[75]
	AA6111	Polyphenylene sulfide (PPS)	[86]
	AA6061	Polyetheretherketone (PEEK)	[82]
	AA6061	Polyamide 6 or Nylon 6 (PA6)	[81]
	AA7075	Polyetheretherketone (PEEK)	[87]
	AMXS6020	MC Nylon 6 (MC PA6)	[80]
	AZ31	Carbon fiber-reinforced polymers (CFRP)	[83]
	AA5058	Poly (methyl methacrylate) (PMMA)	[88]
T-lap joint	AA6062	Poly (methyl methacrylate) (PMMA)	[76]
	AA5754	Poly (methyl methacrylate) (PMMA)	[74]

**Table 4:** Welding tools used in the literature during conventional FSW of metals to polymers.

Tool Type	Shoulder Diameter (mm)	Pin Diameter (mm)	Pin Length (mm)	Reference
Tapered thread pin	20	6	4.2	[75]
	20	8	6.5	[74]
	9	1	1	[77]
	20	4.5	2.5	[85]
	18	5	2.9	[78]
	13.5	5.5	3	[87]
Cylindrical threaded pin	16	5	2.5	[72]
	15	4	2.7	[86]
	16	3	2.8	[79]

**Table 5:** Optimum FSW parameters used to join metals to polymers.

Rotation Speed (rpm)	Traverse Speed (mm/min)	Tilt Angle (degrees)	Plunge Depth (mm)	References
900	50	2	0.2	[82]
710	63	2.5	N/A	[73]
1600	50	2	0.4	[76]
1600	45	2	0.3	[75]
1600	30	2	0.2	[74]
630	30	2.5	0.3	[72]
3250	100	0	0.2	[77]
800	70	3	0.5	[85]
500	40	0	0.5	[78]
1000	40	2	2.8	[79]
1950	2000	4	1.6	[81]
900	60	3	2.3	[80]

**Table 6:** Tensile strength for metal/polymer joint at different velocity indexes. Reprinted with permission from Ref. [78]. 2018, Anjal R. Patel et al.

Specimen Name	Speed (rpm)	Feed (mm/min)	Velocity Index (rev/mm)	Tensile Strength (MPa)
E	500	40	12.50	14.91
K	500	63	7.93	6.02
D	500	80	6.25	5.46
T	710	40	17.75	7.33
J	710	63	11.26	8.78
C	710	80	8.88	5.04
I	1000	40	25.00	5.70
G	1000	63	15.87	7.30
B	1000	80	12.50	8.17
S	1400	40	35.00	4.91
H	1400	63	22.22	5.42
F	1400	80	17.50	8.37

## Data Availability

Not applicable.

## Nomenclature

Al_2_O_3_:	Aluminum oxide
AS:	Advancing side
BM:	Base material
CF-PEEK	Carbon fiber polyetheretherketone
CFRP:	Carbon fiber-reinforced polymers
FSLW:	Friction stir lap welding
FFSW:	Friction fed stir welding
FSW:	Friction stir welding
FFSW:	Friction fed stir welding (FFSW)
HAZ:	Heat affected zone
HDPE:	High-density polyethylene
LDPE:	Low-density polyethylene
MC PA6:	MC Nylon 6
PA6:	Polyamide 6 or Nylon 6
PC:	Polycarbonates
PE:	Polyethylene
PEEK:	Polyetheretherketone
PMMA:	Poly (methyl methacrylate)
PP-C30S:	Polypropylene homopolymer
PPS:	Polyphenylene sulfide
SEM:	Scanning electron microscopy
SiC:	Silicon carbide
SZ:	Stir zone
TMAZ:	Thermo-mechanically affected zone
TT-FSLW:	Top thermic friction stir lap welding
TWI:	The Welding Institute

## References

[1] Sahu S.K., Pal K., Das S. (2020). Parametric study on joint quality in friction stir welding of polycarbonate. Mater. Today Proc..

[2] Arif M., Kumar D., Siddiquee A.N. (2022). Friction stir welding and friction stir spot welding of polymethyl methacrylate (PMMA) to other materials: A review. Mater. Today Proc..

[3] Kah P., Suoranta R., Martikainen J., Magnus C. (2014). Techniques for joining dissimilar materials: Metals and polymers. Rev. Adv. Mater. Sci..

[4] Wang Z., Li C., Sui L., Xian G. (2021). Effects of adhesive property and thickness on the bond performance between carbon fiber reinforced polymer laminate and steel. Thin-Walled Struct..

[5] Heide-Jørgensen S., Møller R.K., Buhl K.B., Pedersen S.U., Daasbjerg K., Hinge M., Budzik M.K. (2018). Efficient bonding of ethylene-propylene-diene M-class rubber to stainless steel using polymer brushes as a nanoscale adhesive. Int. J. Adhes. Adhes..

[6] Han G., Tan B., Cheng F., Wang B., Leong Y.-K., Hu X. (2021). CNT toughened aluminium and CFRP interface for strong adhesive bonding. Nano Mater. Sci..

[7] Cheng F., Hu Y., Zhang X., Hu X., Huang Z. (2021). Adhesive bond strength enhancing between carbon fiber reinforced polymer and aluminum substrates with different surface morphologies created by three sulfuric acid solutions. Compos. Part A: Appl. Sci. Manuf..

[8] Heydari M., Sharif F., Ebrahimi M. (2021). A molecular dynamics study on the role of oxygen-containing functional groups on the adhesion of polymeric films to the aluminum surface. Fluid Phase Equilibria.

[9] Wang J., Zhang G., Zheng X., Li J., Li X., Zhu W., Yanagimoto J. (2020). A self-piercing riveting method for joining of continuous carbon fiber reinforced composite and aluminum alloy sheets. Compos. Struct..

[10] Vignesh N.J. (2021). Analytical Approach for modelling of Heat generation in low-speed friction riveting of polymer/Aluminium joints. Mater. Today Proc..

[11] Hynes N.R.J., Sankaranarayanan R., Sujana J.A.J. (2021). A decision tree approach for energy efficient friction riveting of polymer/metal multi-material lightweight structures. J. Clean. Prod..

[12] Gay A., Lefebvre F., Bergamo S., Valiorgue F., Chalandon P., Michel P., Bertrand P. (2015). Fatigue of Aluminum/Glass Fiber Reinforced Polymer Composite Assembly Joined by Self-piercing Riveting. Procedia Eng..

[13] Staab F., Liesegang M., Balle F. (2020). Local shear strength distribution of ultrasonically welded hybrid Aluminium to CFRP joints. Compos. Struct..

[14] Staab F., Balle F. (2019). Ultrasonic torsion welding of ageing-resistant Al/CFRP joints: Properties, microstructure and joint formation. Ultrasonics.

[15] Mongan P.G., Hinchy E.P., O’Dowd N.P., McCarthy C.T. (2021). Quality prediction of ultrasonically welded joints using a hybrid machine learning model. J. Manuf. Process..

[16] Katayama S., Kawahito Y. (2008). Laser direct joining of metal and plastic. Scr. Mater..

[17] Zhou M., Xiong X., Drummer D., Jiang B. (2019). Interfacial interaction and joining property of direct injection-molded polymer-metal hybrid structures: A molecular dynamics simulation study. Appl. Surf. Sci..

[18] Zhao S., Kimura F., Wang S., Kajihara Y. (2021). Chemical interaction at the interface of metal–plastic direct joints fabricated via injection molded direct joining. Appl. Surf. Sci..

[19] Wang S., Kimura F., Zhao S., Yamaguchi E., Ito Y., Kajihara Y. (2021). Influence of fluidity improver on metal-polymer direct joining via injection molding. Precis. Eng..

[20] Bonpain B., Stommel M. (2018). Influence of surface roughness on the shear strength of direct injection molded plastic-aluminum hybrid-parts. Int. J. Adhes. Adhes..

[21] Lambiase F., Grossi V., Paoletti A. (2022). High-speed joining of hybrid metal-polymer joints during the friction-assisted joining process. Compos. Struct..

[22] Lambiase F., Paoletti A., Durante M. (2021). Mechanism of bonding of AA7075 aluminum alloy and CFRP during friction assisted joining. Compos. Struct..

[23] Lambiase F., Balle F., Blaga L.-A., Liu F., Amancio-Filho S.T. (2021). Friction-based processes for hybrid multi-material joining. Compos. Struct..

[24] Lambiase F., Grossi V., Paoletti A. (2021). Defects formation during Friction Assisted Joining of metals and semi crystalline polymers. J. Manuf. Process..

[25] Lambiase F., Paoletti A. (2018). Mechanical behavior of AA5053/polyetheretherketone (PEEK) made by Friction Assisted Joining. Compos. Struct..

[26] Lambiase F., Paoletti A., Grossi V., Di Ilio A. (2017). Friction assisted joining of aluminum and PVC sheets. J. Manuf. Process..

[27] Liu F.C., Liao J., Nakata K. (2014). Joining of metal to plastic using friction lap welding. Mater. Des..

[28] Braga D.F.O., Eslami S., Moreira P.M.G.P., da Silva L., El-Zein M., Martins P. (2021). Chapter 5—Friction Stir Welding. Advanced Joining Processes.

[29] El-Sayed M.M., Shash A., Abd-Rabou M., ElSherbiny M.G. (2021). Welding and processing of metallic materials by using friction stir technique: A review. J. Adv. Join. Process..

[30] Maji P., Nath R.K., Karmakar R., Paul P., Meitei R.B., Ghosh S.K. (2021). Effect of post processing heat treatment on friction stir welded/processed aluminum based alloys and composites. CIRP J. Manuf. Sci. Technol..

[31] Darras B.M., Omar M., Khraisheh M.K. (2007). Experimental Thermal Analysis of Friction Stir Processing. Mater. Sci. Forum.

[32] Kishta E.E., Darras B. (2014). Experimental investigation of underwater friction-stir welding of 5083 marine-grade aluminum alloy. Proc. Inst. Mech. Eng. Part B: J. Eng. Manuf..

[33] Saad M.H., Darras B.M., Nazzal M.A. (2021). Evaluation of Welding Processes Based on Multi-dimensional Sustainability Assessment Model. Int. J. Precis. Eng. Manuf. Green Technol..

[34] Gabrielli F., Forcellese A., El Mehtedi M., Simoncini M. (2017). Mechanical Properties and Formability of Cold Rolled Friction Stir Welded Sheets in AA5754 for Automotive Applications. Procedia Eng..

[35] Simoncini M., Ciccarelli D., Forcellese A., Pieralisi M. (2014). Micro- and Macro- Mechanical Properties of Pinless Friction Stir Welded Joints in AA5754 Aluminium Thin Sheets. Procedia CIRP.

[36] Bevilacqua M., Ciarapica F.E., D’Orazio A., Forcellese A., Simoncini M. (2017). Sustainability Analysis of Friction Stir Welding of AA5754 Sheets. Procedia CIRP.

[37] Contuzzi N., Campanelli S., Casalino G., Ludovico A. (2016). On the role of the Thermal Contact Conductance during the Friction Stir Welding of an AA5754-H111 butt joint. Appl. Therm. Eng..

[38] Selvaraj M. (2022). Regression model for obtaining peak temperature and heat generation during friction stir welding of stainless steel. Mater. Today Proc..

[39] Cui H., Lu Y., Wang C., Tang X., Liu Z., Misra R. (2022). The occurrence of deformation induced ferrite transition (DIFT) during back heating assisted friction stir welding pipeline steel: The influence on the toughness of welded joint. Mater. Sci. Eng. A.

[40] van Rooyen N., Bhattacharyya M., Charit I., Maughan M.R. (2022). Indentation investigation of 304L stainless steel friction stir weld simulated crack repair. Mater. Sci. Eng. A.

[41] Wang Y., Tsutsumi S., Kawakubo T., Fujii H. (2022). Effects of phosphorus content on fatigue performance of friction stir welded mild steels. Constr. Build. Mater..

[42] Bhatia A., Wattal R. (2020). Friction stir welding of carbon steel: Effect on microstructure and tensile strength. Mater. Today Proc..

[43] Wang Y., Tsutsumi S., Kawakubo T., Fujii H. (2021). Microstructure and mechanical properties of weathering mild steel joined by friction stir welding. Mater. Sci. Eng. A.

[44] Darras B., Kishta E. (2013). Submerged friction stir processing of AZ31 Magnesium alloy. Mater. Des..

[45] Darras B., Khraisheh M., Abu-Farha F., Omar M. (2007). Friction stir processing of commercial AZ31 magnesium alloy. J. Mater. Process. Technol..

[46] Saini S., Chohan J.S., Boparai K.S. (2022). Evaluating the microstructural characteristics in friction stir welding of magnesium AZ61a alloy. Mater. Today Proc..

[47] Chiuzuli F.R., Batistão B.F., Bergmann L.A., de Alcântara N.G., dos Santos J.F., Klusemann B., Gargarella P. (2021). Effect of the gap width in AZ31 magnesium alloy joints obtained by friction stir welding. J. Mater. Res. Technol..

[48] Khalid E., Shunmugasamy V.C., Mansoor B. (2022). Microstructure and tensile behavior of a Bobbin friction stir welded magnesium alloy. Mater. Sci. Eng. A.

[49] Gao F., Guo Y., Yu W., Jiang P., Liao Z. (2021). Microstructure evolution of friction stir welding of Ti6321 titanium alloy based on the weld temperature below microstructure transformation temperature. Mater. Charact..

[50] Gao F., Guo Y., Yang S., Yu Y., Yu W. (2020). Fatigue properties of friction stir welded joint of titanium alloy. Mater. Sci. Eng. A.

[51] Gao F., Guo Y., Qiu S., Yu Y., Yu W. (2020). Fracture toughness of friction stir welded TA5 titanium alloy joint. Mater. Sci. Eng. A.

[52] Nirmal K., Jagadesh T. (2020). Numerical simulations of friction stir welding of dual phase titanium alloy for aerospace applications. Mater. Today Proc..

[53] Sheikh-Ahmad J., Deveci S., Almaskari F., Rehman R.U. (2022). Effect of process temperatures on material flow and weld quality in the friction stir welding of high density polyethylene. J. Mater. Res. Technol..

[54] Sheikh-Ahmad J., Ali D.S., Deveci S., Almaskari F., Jarrar F. (2018). Friction stir welding of high density polyethylene—Carbon black composite. J. Mater. Process. Technol..

[55] Derazkola H.A., Simchi A., Lambiase F. (2019). Friction stir welding of polycarbonate lap joints: Relationship between processing parameters and mechanical properties. Polym. Test..

[56] Derazkola H.A., Garcia E., Elyasi M. (2021). Underwater friction stir welding of PC: Experimental study and thermo-mechanical modelling. J. Manuf. Process..

[57] Derazkola H.A., Simchi A. (2017). Experimental and thermomechanical analysis of friction stir welding of poly(methyl methacrylate) sheets. Sci. Technol. Weld. Join..

[58] Elyasi M., Derazkola H.A. (2018). Experimental and thermomechanical study on FSW of PMMA polymer T-joint. Int. J. Adv. Manuf. Technol..

[59] Argesi F.B., Shamsipur A., Mirsalehi S.E. (2021). Preparation of bimetallic nano-composite by dissimilar friction stir welding of copper to aluminum alloy. Trans. Nonferrous Met. Soc. China.

[60] Kumar R., Kumar G., Roy A., Sinha R.S., Hasnain S.M., Prakash O., Ahmad A. (2022). A comparative analysis of friction stir and tungsten inert gas dissimilar AA5082-AA7075 butt welds. Mater. Sci. Energy Technol..

[61] Fu X., Chen K., Liu C., Wang M., Hua X. (2022). Microstructure and mechanical properties of dissimilar friction stir lap welding between AZ31 Mg alloy and DC01 steel. Mater. Charact..

[62] Sahu S., Mypati O., Pal S.K., Shome M., Srirangam P. (2021). Effect of weld parameters on joint quality in friction stir welding of Mg alloy to DP steel dissimilar materials. CIRP J. Manuf. Sci. Technol..

[63] Geng P., Morimura M., Ma H., Ma Y., Ma N., Liu H., Aoki Y., Fujii H., Qin G. (2022). Elucidation of intermetallic compounds and mechanical properties of dissimilar friction stir lap welded 5052 Al alloy and DP590 steel. J. Alloy. Compd..

[64] Do H., Asadi S., Park N. (2022). Microstructural and mechanical properties of dissimilar friction stir welded CoCrFeMnNi high entropy alloy to STS304 stainless steel. Mater. Sci. Eng. A.

[65] Ke W., Oliveira J., Ao S., Teshome F., Chen L., Peng B., Zeng Z. (2022). Thermal process and material flow during dissimilar double-sided friction stir spot welding of AZ31/ZK60 magnesium alloys. J. Mater. Res. Technol..

[66] Zhao Y., Luo Y., Lu Y., He Y., Guo X., Wang S., Cui H., Zhang Y., Wang Z. (2021). Effect of welding parameters on the microstructures and mechanical properties of double-pass aluminum/magnesium dissimilar metal friction stir lap welding joint. Mater. Today Commun..

[67] Sundar A.S., Vardhan T.V., Kumar A. (2022). Microstructural characterization of aluminium-titanium friction stir welds. Mater. Today Proc..

[68] Kar A., Malopheyev S., Mironov S., Kaibyshev R., Suwas S., Kailas S.V. (2021). A new method to elucidate fracture mechanism and microstructure evolution in titanium during dissimilar friction stir welding of aluminum and titanium. Mater. Charact..

[69] Hajideh M.R., Farahani M., Alavi S.A.D., Ramezani N.M. (2017). Investigation on the effects of tool geometry on the microstructure and the mechanical properties of dissimilar friction stir welded polyethylene and polypropylene sheets. J. Manuf. Process..

[70] Kumar S., Roy B.S. (2019). Novel study of joining of acrylonitrile butadiene styrene and polycarbonate plate by using friction stir welding with double-step shoulder. J. Manuf. Process..

[71] Sidhom A.A., Naga S.A., Kamal A. (2022). Friction stir spot welding of similar and dissimilar high density polyethylene and polypropylene sheets. Adv. Ind. Manuf. Eng..

[72] MirHashemi S., Amadeh A., Khodabakhshi F. (2021). Effects of SiC nanoparticles on the dissimilar friction stir weldability of low-density polyethylene (LDPE) and AA7075 aluminum alloy. J. Mater. Res. Technol..

[73] Khodabakhshi F., Haghshenas M., Sahraeinejad S., Chen J., Shalchi B., Li J., Gerlich A. (2014). Microstructure-property characterization of a friction-stir welded joint between AA5059 aluminum alloy and high density polyethylene. Mater. Charact..

[74] Derazkola H.A., Simchi A. (2019). An investigation on the dissimilar friction stir welding of T-joints between AA5754 aluminum alloy and poly(methyl methacrylate). Thin-Walled Struct..

[75] Derazkola H.A., Elyasi M. (2018). The influence of process parameters in friction stir welding of Al-Mg alloy and polycarbonate. J. Manuf. Process..

[76] Derazkola H.A., Simchi A. (2020). A new procedure for the fabrication of dissimilar joints through injection of colloidal nanoparticles during friction stir processing: Proof concept for AA6062/PMMA joints. J. Manuf. Process..

[77] Rahmat S.M., Hamdi M., Yusof F., Moshwan R. (2014). Preliminary study on the feasibility of friction stir welding in 7075 aluminium alloy and polycarbonate sheet. Mater. Res. Innov..

[78] Patel A.R., Kotadiya D.J., Kapopara J.M., Dalwadi C.G., Patel N.P., Rana H. (2018). Investigation of Mechanical Properties for Hybrid Joint of Aluminium to Polymer using Friction Stir Welding (FSW). Mater. Today Proc..

[79] Dalwadi C.G., Patel A.R., Kapopara J.M., Kotadiya D.J., Patel N.D., Rana H. (2018). Examination of Mechanical Properties for Dissimilar Friction Stir Welded Joint of Al Alloy (AA-6061) to PMMA (Acrylic). Mater. Today Proc..

[80] Gao Y., Morisada Y., Fujii H., Liao J. (2019). Friction stir lap welding of plastic to metal using adjustable tool. Sci. Technol. Weld. Join..

[81] Wang W., Wang S., Zhang X., Xu Y., Tian Y., Huang H. (2021). Enhanced aluminum alloy-polymer friction stir welding joints by introducing micro-textures. Mater. Lett..

[82] Huang Y., Meng X., Wang Y., Xie Y., Zhou L. (2018). Joining of aluminum alloy and polymer via friction stir lap welding. J. Mater. Process. Technol..

[83] Wang T., Li L., Pallaka M.R., Das H., Whalen S., Soulami A., Upadhyay P., Kappagantula K.S. (2021). Mechanical and microstructural characterization of AZ31 magnesium-carbon fiber reinforced polymer joint obtained by friction stir interlocking technique. Mater. Des..

[84] Dong H., Tang Z., Li P., Wu B., Hao X., Ma C. (2021). Friction stir spot welding of 5052 aluminum alloy to carbon fiber reinforced polyether ether ketone composites. Mater. Des..

[85] Shahmiri H., Movahedi M., Kokabi A.H. (2017). Friction stir lap joining of aluminium alloy to polypropylene sheets. Sci. Technol. Weld. Join..

[86] Ratanathavorn W., Melander A. (2015). Dissimilar joining between aluminium alloy (AA 6111) and thermoplastics using friction stir welding. Sci. Technol. Weld. Join..

[87] Li M., Xiong X., Ji S., Hu W., Yue Y. (2021). Achieving high-quality metal to polymer-matrix composites joint via top-thermic solid-state lap joining. Compos. Part B: Eng..

[88] Derazkola H.A., Fard R.K., Khodabakhshi F. (2018). Effects of processing parameters on the characteristics of dissimilar friction-stir-welded joints between AA5058 aluminum alloy and PMMA polymer. Weld. World.

[89] Nandan R., DebRoy T., Bhadeshia H. (2008). Recent advances in friction-stir welding—Process, weldment structure and properties. Prog. Mater. Sci..

[90] Harisha P., Nanjundaswamy H., Divakar H., Krishnan D. (2021). Tensile properties of aluminium and copper alloys friction stir welded joints. Mater. Today Proc..

[91] Kumar M., Das A., Ballav R. (2020). Influence of tool geometry on morphology and mechanical properties of friction stir welded dissimilar joints: A review. Mater. Today Proc..

[92] Haghshenas M., Khodabakhshi F. (2019). Dissimilar friction-stir welding of aluminum and polymer: A review. Int. J. Adv. Manuf. Technol..

[93] Mishra R.S., Ma Z.Y. (2005). Friction stir welding and processing. Mater. Sci. Eng. R Rep..

[94] Huang Y., Meng X., Xie Y., Wan L., Lv Z., Cao J., Feng J. (2018). Friction stir welding/processing of polymers and polymer matrix composites. Compos. Part A: Appl. Sci. Manuf..

[95] Meilinger Á., Török I. (2013). The importance of friction stir welding tool. Prod. Processes Syst..

[96] Payganeh G.H., Arab N.B.M., Asl Y.D., Ghasemi F.A., Boroujeni M.S. (2011). Effects of friction stir welding process parameters on appearance and strength of polypropylene composite welds. Int. J. Phys. Sci..

